# Supplementary material for: Susceptibility and infectiousness of SARS-CoV-2 in children versus adults, by variant (wild-type, alpha, delta): A systematic review and meta-analysis of household contact studies
Source: PLoS One. 2024 Sep 6;19(9):e0306740. doi: 10.1371/journal.pone.0306740 (PMC11379298; doi:10.1371/journal.pone.0306740)
Supplement: S1 File — (DOCX) [file pone.0306740.s003.docx]

**Online Only Figures**

**eFigure 1: Forest plot for susceptibility showing secondary attack rates among children, adolescents and adults for wild-type.**

**
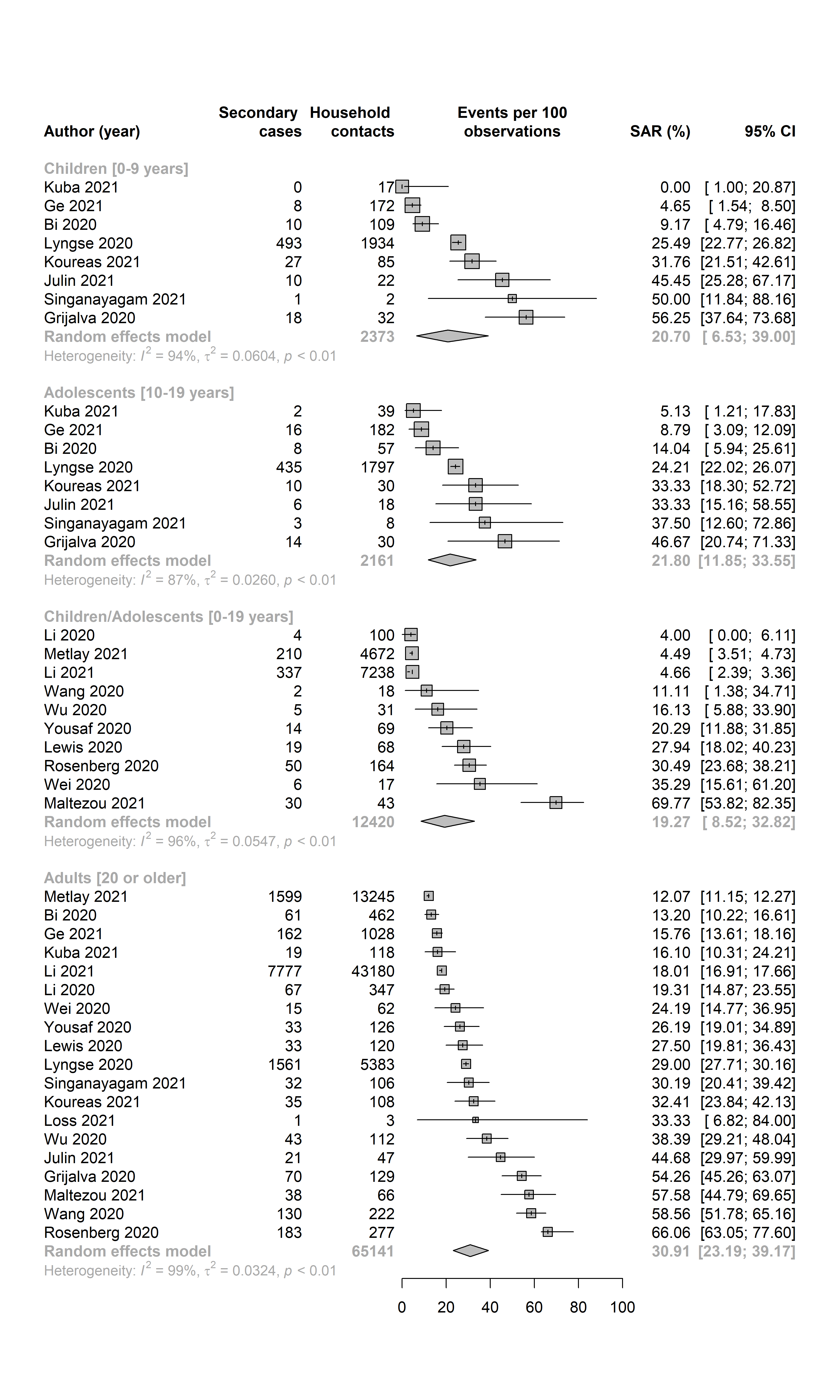
**

**eFigure 2: Forest plot for susceptibility showing secondary attack rates among children, adolescents, and adults for Alpha**

**
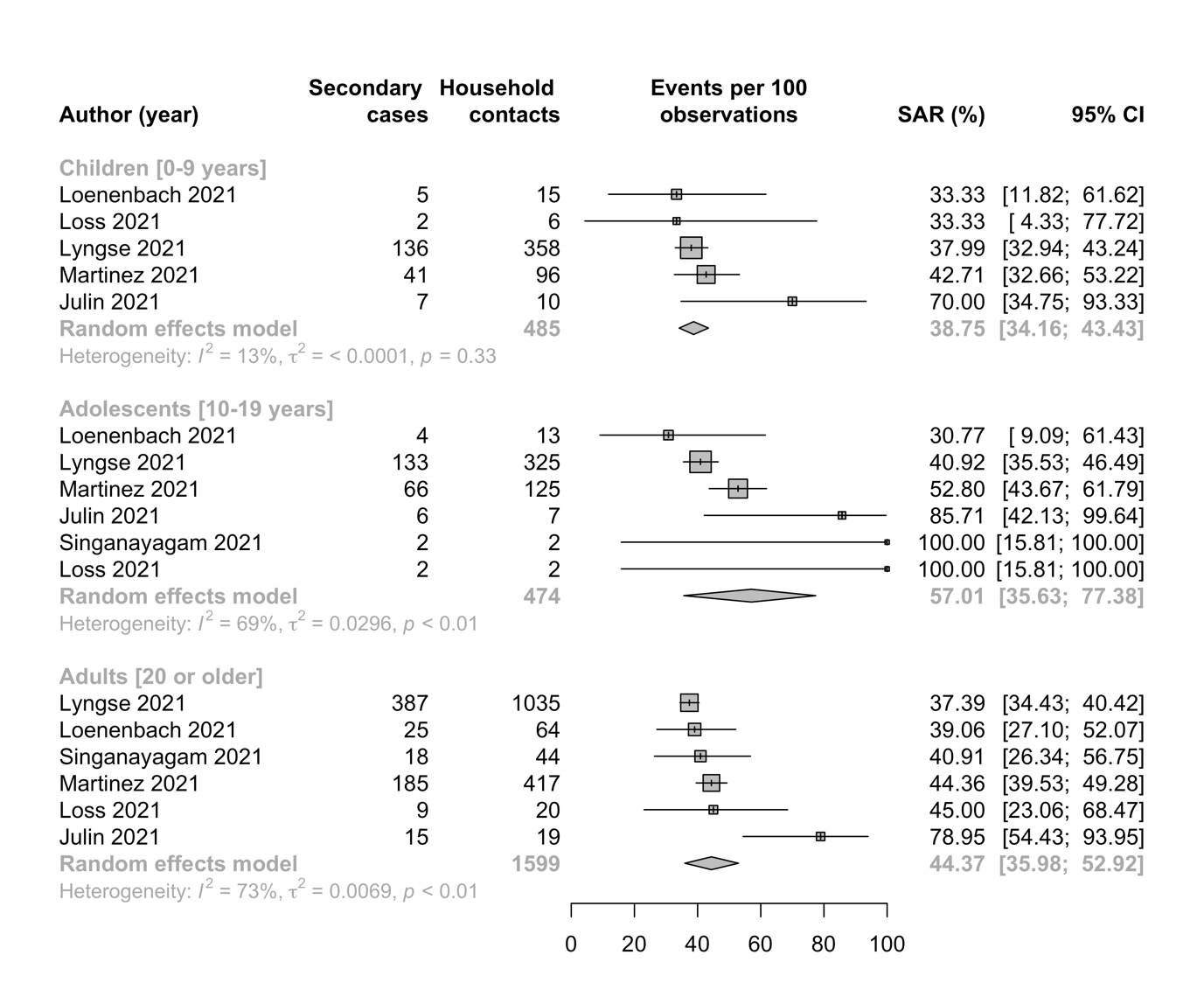
**

**eFigure 3: Forest plot for susceptibility showing secondary attack rates among children, adolescents, and adults for Delta**

**
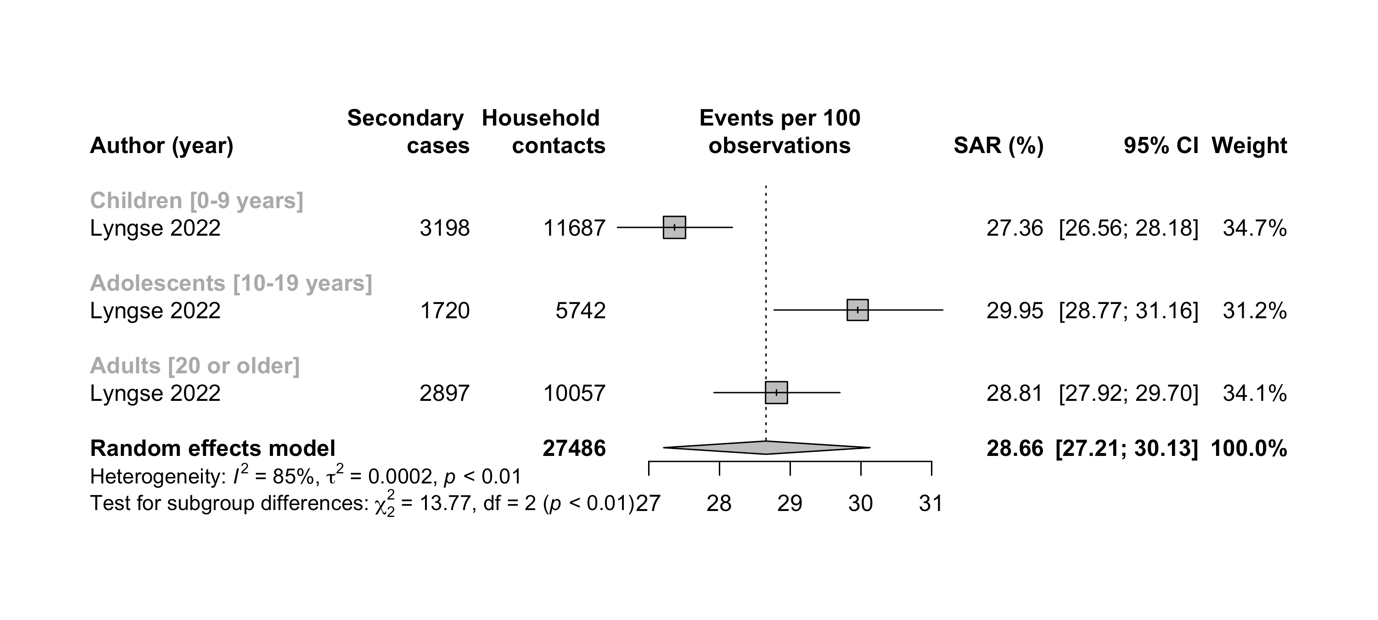
**

**eFigure 4: Forest plot for infectiousness showing secondary attack rates when children, adolescents and adults were index cases and infected with wild-type SARS-CoV-2.**

**
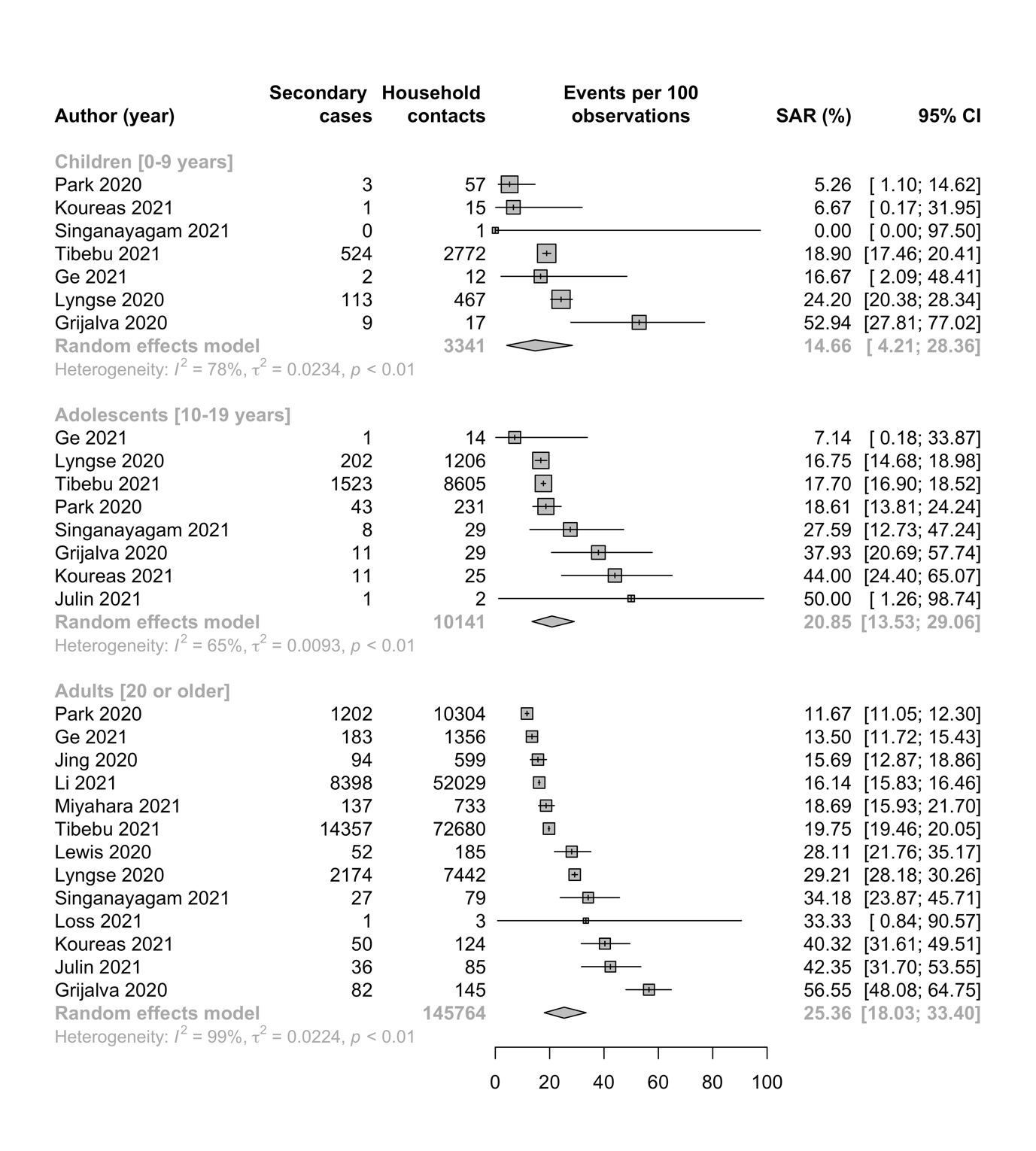
**

**eFigure 5: Forest plot for infectiousness showing secondary attack rates when children, adolescents and adults were index cases and infected with Alpha variant of SARS-CoV-2.**

**
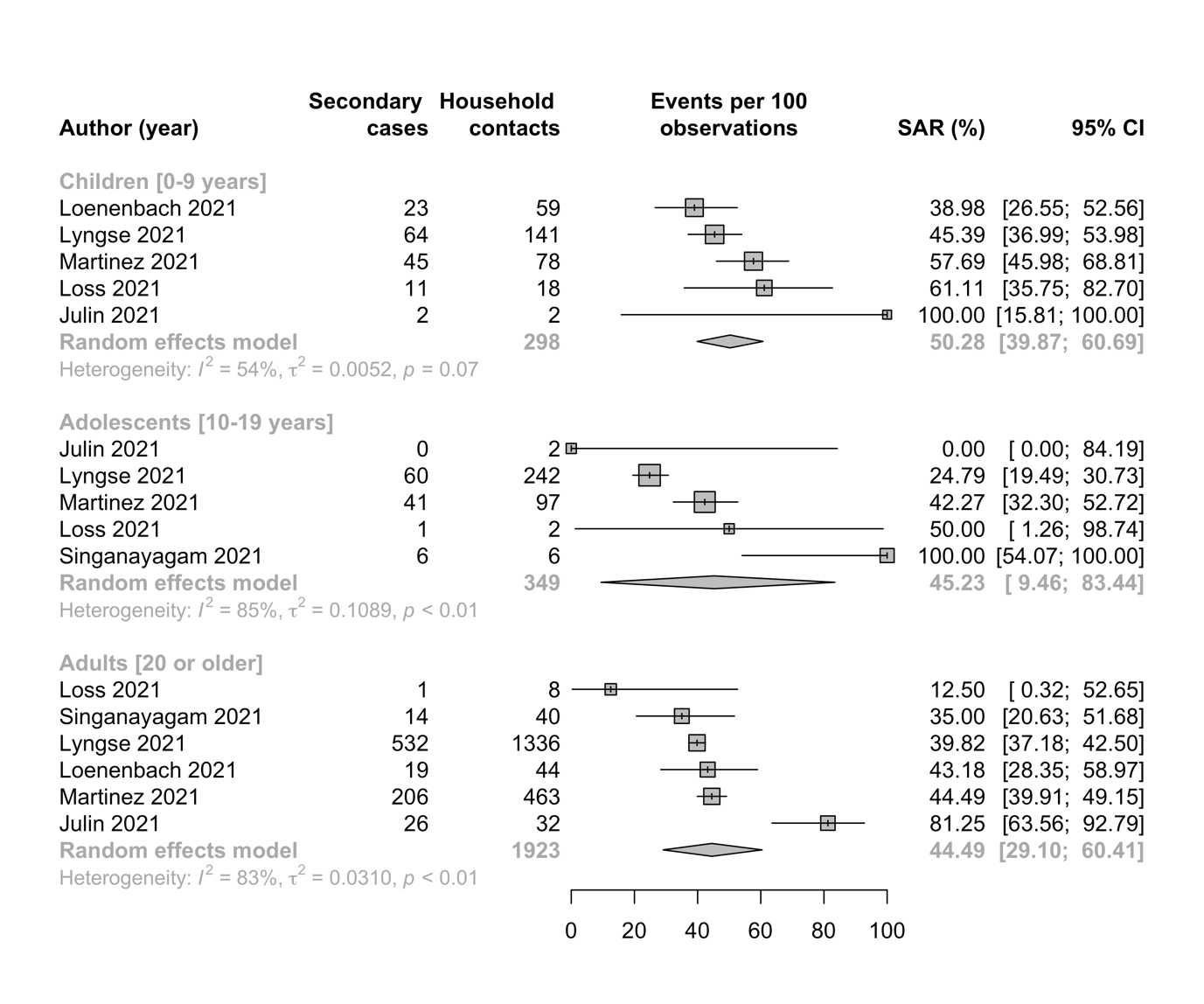
**

**eFigure 6: Forest plot for infectiousness showing secondary attack rates among children, adolescents, and adults for Delta**


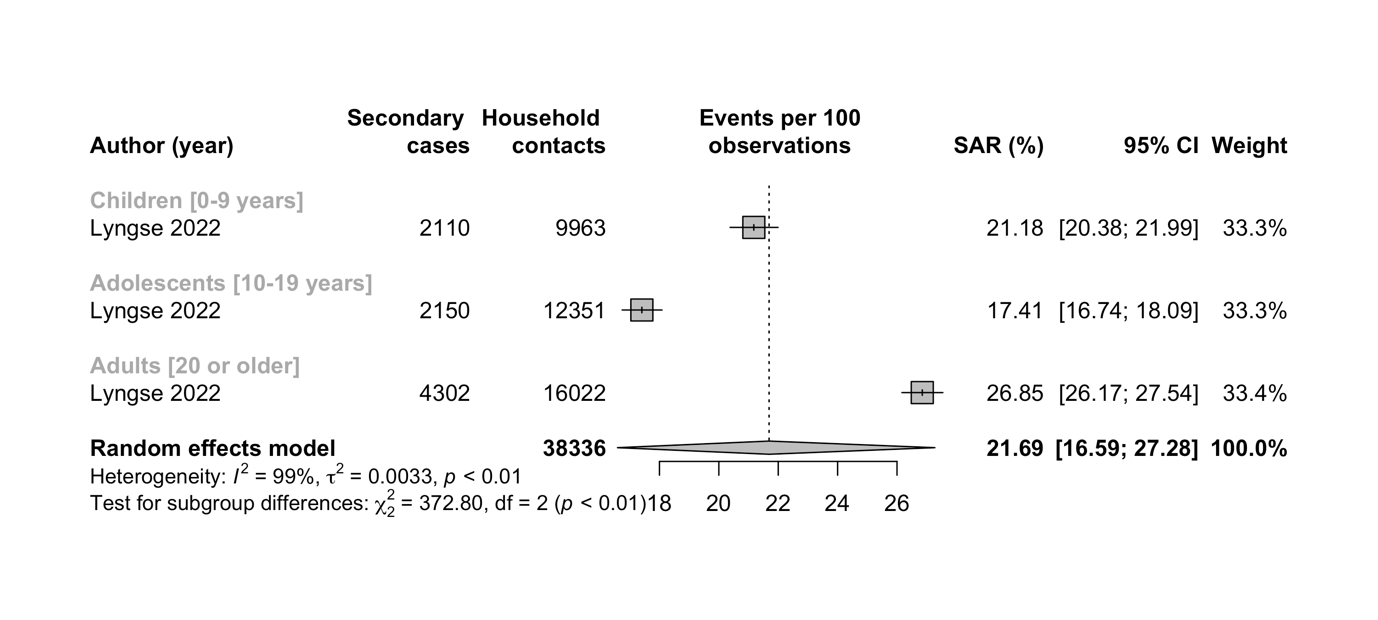


**eTable 1: Summary characteristics of included studies**

| **Study** | **Country of study** | **Study design:** | **Variant type** | **Study period:** | **No of index cases** | **No of contact identified** | **Household SAR (%)** | **Mean age (yrs)** | **% Female** | **Number of tests per contact** | **Follow up duration (days)** | **Case identification** | Case definition index case | Case definition secondary case | Vaccination |
| --- | --- | --- | --- | --- | --- | --- | --- | --- | --- | --- | --- | --- | --- | --- | --- |
| Bi 2020^1^ | China | Retrospective cohort | Wild-type | January 14 to February 12, 2020 | 397 | 1,286 | 11.2 | 45 | 48 | Multiple | 12 plus | Real-time reverse transcription polymerase chain reaction (RT-PCR) of nasal swabs | Community cases | Close contacts were identified through contact tracing of a confirmed case and were defined as those who lived in the same apartment, shared a meal, travelled, or socially interacted with an index case 2 days before symptom onset | Likely all households unvaccinated |
| Ge 2021^2^ | China | Prospective cohort | Wild-type | January 8 to July 30, 2020 | 730 | 8,852 | 10.1 | 46 | 48.8 | Multiple | 14 | RT-PCR using respiratory specimens | Community cases | Active symptomatic follow-up | Likely all households unvaccinated |
| Grijalva 2020^3^ | US | Surveillance data | Wild-type | April–September 2020 | 101 | 191 | 53 | 28 | 54 | Multiple | 14 | RT-PCR of nasal swabs or saliva specimens | Community cases | Active symptomatic follow-up | Likely all households unvaccinated |
| Jing 2020^4^ | China | Retrospective cohort | Wild-type | Jan 7, 2020, and Feb 18, 2020 | 349 | 195 | 12.4 | Not provided | 50 | Multiple | 14 | RT-PCR of nasal swabs | Community cases | Passive identification of secondary cases independent from symptoms | Likely all households unvaccinated |
| Julin 2021^5^ | Norway | Prospective cohort | Wild-type, Alpha | May 2020 to May 2021 | 65 | 200 | 49.6 | 31 | 52 | Multiple | 42 | RT-PCR of Oropharyngeal (OP) samples and neat saliva samples | A >=12 yo case in a household with the first positive test result (“primary case”) and where no other household contact person tested positive on the same day. | Households with vaccinated individuals not eligible. All hhc >=2 years were tested several times until day 14 after test date of primary case irrespective of symptoms. | Households with vaccinated individuals excluded. |
| Koureas 2021^6^ | Greece | Surveillance data | Wild-type | 8 April to 4 June 2020 | 142 | 135 | 38.62 | Not provided | Not provided | Multiple | 14 | RT-PCR of oropharyngeal samples | Community cases | Passive identification of secondary cases independent from symptoms | Likely all households unvaccinated |
| Kuba 2021^7^ | Japan | Surveillance data | Wild-type | February 14 to May 31, 2020 | 78 | 174 | 12.1 | 51.5 | 32 | 1 | 14 | RT-PCR of throat and nasopharyngeal swaps | Community cases | Passive identification of secondary cases independent from symptoms | Likely all households unvaccinated |
| Lewis 2020^8^ | US | Surveillance data | Wild-type | 4 months | 575,071 | 62 | 29 | Not provided | Not provided | Multiple | 14 | RT-PCR of nasopharyngeal or anterior nasal swabs | Community cases | Passive identification of secondary cases independent from symptoms | Likely all households unvaccinated |
| Li 2020^9^ | China | Retrospective cohort | Wild-type | January 1 to February 20, 2020 | 105 | 392 | 18 | 47 | 43.9 | Multiple | 14 | RT-PCR of nasopharyngeal swaps | Community cases | Passive identification of secondary cases independent from symptoms | Likely all households unvaccinated |
| Li 2021^10^ | China | Retrospective cohort | Wild-type | Dec 2, 2019 to April 18, 2020 | 27,101 | 27,101 | 15·6 | 56 | 48 | Not provided | No clearly stated | RT-PCR of respiratory specimens | Community cases | Passive identification of secondary cases independent from symptoms | Likely all households unvaccinated |
| Loenenbach 2021^11^ | Germany | Retrospective cohort | Alpha | 2021 | Not provided | 92 | 37 | Not provided | Not provided | 1 | 14 | PCR test | Cases of an outbreak in a childcare centre | Passive identification of secondary cases independent from symptoms. | Likely all households unvaccinated |
| Loss, 2021^12^ | Germany | Cohort | Alpha, Wild-type | October 2020 to June 2021 | 33 | 45 | 53.3 | Not provided | 61 | Multiple | 12 | RT-PCR | Cases recruited in a study in childcare centres | Active follow-up and systematic, serial testing of household contacts irrespective of symptoms | Adults were asked about their vaccination status in a telephone interview. None of the adults were fully vaccinated (5 individuals in the household cohort had received 1 of 2 shots shortly before the home visits). |
| Lyngse 2020^13^ | Denmark | Register data | Wild-type | February - July 24, 2020 | 6,782 | 6,609 | 17 | Not provided | Not provided | Multiple | 14 | RT-PCR | Primary case was first positive test for SARS-CoV-2. | Secondary cases were defined as those who had a positive test within 14 days of the primary case being tested positive. | Likely all households unvaccinated |
| Lyngse 2021^14^ | Denmark | Register data | Alpha | January 11 to February 7, 2021 | 8,093 | 16,612 | 25 |  | 45 | Multiple | 14 | RT-PCR | Primary case was first positive test for SARS-CoV-2. | Secondary cases were defined as those who had a positive test within 14 days of the primary case being tested positive. | Likely all households unvaccinated |
| Lyngse 2022^15^ | Denmark | Register data | Delta | June 21 to October 25, 2021 | 24,693 | 16,612 | 25 |  | 45 | Multiple | 14 | RT-PCR | The case in a household with the first positive test result (“primary case”) and where no other household contact person tested positive on the same day. | Household contact persons testing positive 1-14 days after test date of the primary case. No information provided if testing based on symptoms or recommended on all household contact persons. More than 70% of all hhc were tested. | Documented and analysed separately for vaccinated/unvaccinated index/primary and hhc. |
| Maltezoua 2021^16^ | Greece | Surveillance data | Wild-type | Febrary 26-May 3, 2020 | 109 household | 109 | 8.8 | Not provided | Not provided | Not stated | 14 | RT-PCR on respiratory specimens | Community cases | Passive identification of secondary household cases independent from symptoms. | Likely all household unvaccinated |
| Martinez 2021^17^ | US | Cohort | Alpha | June 11, 2020 to May 20, 2021 | 277 | 638 | 45.8 | 29.8 | Not provided | 1 | 14 | RT-PCR | Community cases | Passive identification of secondary household cases independent from symptoms. | Likely all household unvaccinated |
| Metlay 2021^18^ | US | Retrospective cohort | Wild-type | March 4 - May 17, 2020 | 7,262 | 17,917 | Not stated | Not provided | 47.8 | Not stated | Not provided | RT-PCR | Community cases | Passive identification of secondary household cases independent from symptoms. | Likely all household unvaccinated |
| Miyahara, 2021^19^ | Japan | Cohort | Wild-type | February 22-May 31, 202 | 306 | 147 | 19 | Not provided | 49.7 | Not provided | Not provided | RT-PCR | Community cases | Passive identification of secondary cases independent from symptoms | Likely all households unvaccinated |
| Park 2020^20^ | South Korea | Surveillance data | Wild-type | January 20 to May 13 2020 | 5,706 | 59,073 | 11.8 | Not provided | Not provided | Multiple | 14 | RT-PCR | Community cases | Passive identification of secondary cases independent from symptoms | Likely all households unvaccinated |
| Rosenberg 2020^21^ | US | Surveillance data | Wild-type | March 2 – March 31, 2020 | 229 |  | Not stated | 43 | 43.7 |  |  | RT-PCR of throat swabs | Community cases | Passive identification of secondary cases independent from symptoms | Likely all households unvaccinated |
| Singanayagam 2021^22^ | UK | Prospective cohort | Wild-type, Alpha, Delta | September 13, 2020 to September 15, 2021 | 471 | 602 | 25 | 36 | 55 | Multiple | 14-20 | RT-PCR of throat swabs | Community cases | Active symptomatic follow-up, all contacts notified within 5 days of index case symptom | Fully vaccinated and unvaccinated individuals |
| Tibebu 2021^23^ | Canada | Surveillance data | Wild-type | July 1 to November 30, 2020 | 29,352 | 84,125 | 19.5 | 44 | 19 | 1 | 14-28 | RT-PCR | Community cases | Passive identification of secondary cases independent from symptoms | Likely all households unvaccinated |
| Wang, 2020^24^ | China | Retrospective case series | Wild-type | February 13-28 | 20,399 | 155 | 24 | Not provided | 48 | Multiple | Not stated | RT-PCR on throat swabs | Community cases | Passive identification of secondary cases independent from symptoms | Likely all households unvaccinated |
| Wei 2020^25^ | China | Surveillance data | Wild-type | Jan 1 to Feb 14, 2020 | 23 | 79 | 52 | 33.9 | 58.3 | Not stated | Not stated | Not provided | Community cases | Passive identification of secondary cases independent from symptoms | Likely all households unvaccinated |
| Wu, 2020^26^ | China | Surveillance data | Wild-type | January –February 2020 | 46 | 104 | 32.4 | 43.8 | Not provided | Not stated | 21 | RT-PCR of nasopharyngeal and/or oropharyngeal swabs | Community cases | Passive identification of secondary cases independent from symptoms | Likely all households unvaccinated |
| Yousaf 2020^27^ | US | Prospective cohort | Wild-type | 22 March to 22 April 2020 | 198 | 47 | Not stated | 24 | 62 | Multiple | 14 | RT-PCR of nasopharyngeal swab | Community cases | Active symptomatic follow-up | Likely all households unvaccinated |

**eTable 2: Risk of bias of included studies**

|  | **Representativeness of the index cases in region (2 points)^a^** | **Index case definition (1 point)^b^** | **Sample size (1 point)^c^** | **Household secondary attack rate disaggregated by index / contact age (1 point)^d^** | **Universal or symptomatic based testing (1 point)^e^** | **Follow-up duration ( 2 points)^f^** | **Number of test per contact (1 point)^g^** | **Total points** | **Risk of bias^h^** |
| --- | --- | --- | --- | --- | --- | --- | --- | --- | --- |
| Bi 2020^1^ | ++ | + | + | + | + | + | + | 8 | Low |
| Ge 2021^2^ | ++ | + | + | + | + | ++ | + | 9 | Low |
| Grijalva 2020^3^ | + | + | + | + | + | ++ | + | 8 | Low |
| Jing 2020^4^ | ++ | + | + | + | + | ++ | + | 9 | Low |
| Julin 2021^28^ | + | + | 0 | + | + | ++ | + | 7 | Low |
| Koureas 2021^6^ | ++ | + | + | + | + | ++ | + | 9 | Low |
| Kuba 2021^7^ | + | + | 0 | + | + | ++ | 0 | 6 | Moderate |
| Lewis 2020^8^ | ++ | + | + | + | + | ++ | + | 9 | Low |
| Li 2020^9^ | + | + | 0 | + | + | ++ | + | 7 | Low |
| Li 2021^10^ | ++ | + | + | + | + | 0 | 0 | 6 | Moderate |
| Loenenbach 2021^11^ | + | + | 0 | + | + | ++ | 0 | 6 | Moderate |
| Loss, 2021^12^ | ++ | + | 0 | + | + | + | + | 7 | Moderate |
| Lyngse 2020^13^ | ++ | + | + | + | + | ++ | + | 9 | Low |
| Lyngse 2021^14^ | ++ | + | + | + | + | ++ | + | 9 | Low |
| Lyngse 2022^15^ | ++ | + | + | + | + | ++ | + | 9 | Low |
| Maltezoua 2021^16^ | + | + | + | + | + | ++ | 0 | 7 | Low |
| Martinez 2021^17^ | ++ | + | + | + | + | ++ | 0 | 8 | Low |
| Metlay 2021^18^ | ++ | + | + | + | + | 0 | 0 | 6 | Moderate |
| Miyahara, 2021^19^ | + | 0 | + | + | + | 0 | + | 5 | Moderate |
| Park 2020^20^ | ++ | + | + | + | + | ++ | + | 9 | Low |
| Rosenberg 2020^21^ | ++ | + | 0 | + | + | 0 | 0 | 5 | Moderate |
| Singanayagam 2021^22^ | + | + | + | + | + | ++ | + | 8 | Low |
| Tibebu 2021^23^ | ++ | + | + | + | + | ++ | 0 | 8 | Low |
| Wang, 2020^24^ | ++ | + | + | + | + | 0 | + | 7 | Low |
| Wei 2020^25^ | ++ | + | 0 | + | + | 0 | 0 | 5 | Moderate |
| Wu, 2020^26^ | + | + | 0 | + | + | ++ | 0 | 6 | moderate |
| Yousaf 2020^27^ | ++ | + | 0 | + | + | ++ | + | 8 | Low |

a ++: Representative of COVID-19 cases in region; +: Somewhat representative; 0: Poorly described or not representative of cases in region

b +: Index case identified by date of onset of symptoms and/or test dates; 0: First case not clearly defined

c +: ≥300 contacts; 0: <300 contacts

d +: Secondary attack rate disaggregated by ≥1 covariate; 0: Secondary attack rate not disaggregated by any covariates

e +: Tested all contacts (both symptomatic and asymptomatic); 0: Only tested symptomatic contacts

f ++: >14 days; +: 14 days; 0: <14 days or not specified

g +: ≥2 tests; 0: 1 test or not described

h High: ≤3 points; moderate: 4–6 points; low: ≥7 points

**Annex 1: Search strategy**

| SARS-CoV-2/ or COVID-19/  (corona* adj1 (virus* or viral*)).mp.  (CoV not (Coefficien* or "co-efficien*" or covalent* or Covington* or covariant* or covarianc* or "cut-off value*" or "cutoff value*" or "cut-off volume*" or "cutoff volume*" or "combined optimi?ation value*" or "central vessel trunk*" or CoVR or CoVS)).mp.  (coronavirus* or 2019nCoV* or 19nCoV* or "2019 novel*" or Ncov* or "n-cov" or "SARS- CoV-2*" or "SARSCoV-2*" or SARSCoV2* or "SARS-CoV2*" or "severe acute respiratory syndrome*" or COVID*2).mp.  "Severe Acute Respiratory Syndrome Coronavirus 2".mp.  "COVID-19".mp.  "covid 19 diagnostic testing"2.mp.  "covid 19 drug treatment".mp.  "covid 19 serotherapy".mp.  "covid 19 vaccine".mp.  ncov*.mp.  covid*.mp.  sars-cov-2.mp.  "sars cov 2".mp.  "SARS Coronavirus 2".mp.  "Severe Acute Respiratory Syndrome CoV 2".mp.  "Wuhan coronavirus".mp.  "Wuhan seafood market pneumonia virus".mp.  "SARS2".mp.  "2019-nCoV".mp.  "hcov-19".mp.  "novel 2019 coronavirus".mp.  "2019 novel coronavirus*".mp.  "novel coronavirus 2019*".mp.  "2019 novel human coronavirus*".mp.  "human coronavirus 2019".mp.  "coronavirus disease-19".mp.  "corona virus disease-19".mp.  "coronavirus disease 2019".mp.  "corona virus disease 2019".mp.  "2019 coronavirus disease".mp.  "2019 corona virus disease".mp.  "novel coronavirus disease 2019".mp.  "novel coronavirus infection 2019".mp.  "new coronavirus*".mp.  "coronavirus outbreak".mp.  "coronavirus epidemic".mp.  "coronavirus pandemic".mp.  "pandemic of coronavirus".mp.  or/1-39  age.mp.  "age group*".mp.  Children*.mp.  child.mp.  childhood.mp.  teen*.mp.  pediatric*.mp.  paediatric*.mp.  adolescen*.mp.  boys.mp.  girls.mp.  youth.mp.  youths.mp.  or/41-53  (variant* or mutant* or mutation* or strain*).mp.  (alpha or S-gene target failure or SGTF or beta or gamma or delta or epsilon or zeta or eta or theta or iota or kappa or lambda).mp.  ("B.1.1.7" or "20I/501Y.V1" or "VOC 202012/01" or "B.1.351" or "20H/501Y.V2" or "P.1" or "20J/501Y.V3" or "B.1.1.28" or "501Y*" or "N501Y" or "E484K" or "D614G" or "69/70 deletion" or "144Y deletion" or "A570D" or "P681H" or "K417N*" or "VUI-202012/01" or "Kent" or "B.1.427" or "CA VUI1" or "CAL.20C" or "B.1.429" or "B.1.526" or "B.1.525" or "P.2" or "P.3" or "B.1.617" or "B.1.617.2" or "C.37").mp.  or/55-57  transmission.mp.  disease susceptibility.mp.  communicability.mp.  contagious.mp.  contagiousness.mp.  susceptibility.mp.  epidemiology.mp.  "contact tracing".mp.  "communicable disease contact tracing".mp.  infection.mp.  infectious*.mp.  "attack rate".mp.  “household”.mp.  "secondary attack rate".mp.  or/59-72  (shed* or viabl*).mp.  (viral or virus or rna or ribonucleic).mp.  viral clearance.mp.  viral load.mp.  viral shedding.mp.  or/74-78 |
| --- |

1. Bi Q, Wu Y, Mei S, et al. Epidemiology and transmission of COVID-19 in 391 cases and 1286 of their close contacts in Shenzhen, China: a retrospective cohort study. *Lancet Infect Dis* 2020;20(8):911-19. doi: 10.1016/s1473-3099(20)30287-5 [published Online First: 20200427]

2. Ge Y, Martinez L, Sun S, et al. COVID-19 Transmission Dynamics Among Close Contacts of Index Patients With COVID-19: A Population-Based Cohort Study in Zhejiang Province, China. *JAMA Intern Med* 2021;181(10):1343-50. doi: 10.1001/jamainternmed.2021.4686

3. Grijalva CG, Rolfes MA, Zhu Y, et al. Transmission of SARS-COV-2 Infections in Households - Tennessee and Wisconsin, April-September 2020. *MMWR Morb Mortal Wkly Rep* 2020;69(44):1631-34. doi: 10.15585/mmwr.mm6944e1 [published Online First: 20201106]

4. Jing QL, Liu MJ, Zhang ZB, et al. Household secondary attack rate of COVID-19 and associated determinants in Guangzhou, China: a retrospective cohort study. *Lancet Infect Dis* 2020;20(10):1141-50. doi: 10.1016/s1473-3099(20)30471-0 [published Online First: 20200617]

5. Julin CH, Robertson AH, Hungnes O, et al. Household Transmission of SARS-CoV-2: A Prospective Longitudinal Study Showing Higher Viral Load and Increased Transmissibility of the Alpha Variant Compared to Previous Strains. *Microorganisms* 2021;9(11):2371.

6. Koureas M, Speletas M, Bogogiannidou Z, et al. Transmission Dynamics of SARS-CoV-2 during an Outbreak in a Roma Community in Thessaly, Greece-Control Measures and Lessons Learned. *Int J Environ Res Public Health* 2021;18(6) doi: 10.3390/ijerph18062878 [published Online First: 20210311]

7. Kuba Y, Shingaki A, Nidaira M, et al. The characteristics of household transmission during COVID-19 outbreak in Okinawa, Japan from February to May 2020. *Jpn J Infect Dis* 2021 doi: 10.7883/yoken.JJID.2020.943 [published Online First: 20210430]

8. Lewis NM, Chu VT, Ye D, et al. Household Transmission of Severe Acute Respiratory Syndrome Coronavirus-2 in the United States. *Clin Infect Dis* 2021;73(7):1805-13. doi: 10.1093/cid/ciaa1166

9. Li W, Zhang B, Lu J, et al. Characteristics of Household Transmission of COVID-19. *Clin Infect Dis* 2020;71(8):1943-46. doi: 10.1093/cid/ciaa450

10. Li F, Li YY, Liu MJ, et al. Household transmission of SARS-CoV-2 and risk factors for susceptibility and infectivity in Wuhan: a retrospective observational study. *Lancet Infect Dis* 2021;21(5):617-28. doi: 10.1016/s1473-3099(20)30981-6 [published Online First: 20210118]

11. Loenenbach A, Markus I, Lehfeld AS, et al. SARS-CoV-2 variant B.1.1.7 susceptibility and infectiousness of children and adults deduced from investigations of childcare centre outbreaks, Germany, 2021. *Euro Surveill* 2021;26(21) doi: 10.2807/1560-7917.Es.2021.26.21.2100433

12. Loss J, Wurm J, Varnaccia G, et al. Transmission of SARS-CoV-2 among children and staff in German daycare centres. *Epidemiology and Infection* 2022:1-25. doi: 10.1017/S0950268822001194 [published Online First: 2022/07/08]

13. Lyngse FP, Kirkeby CT, Halasa T, et al. COVID-19 Transmission Within Danish Households: A Nationwide Study from Lockdown to Reopening. *medRxiv* 2020:2020.09.09.20191239. doi: 10.1101/2020.09.09.20191239

14. Lyngse FP, Mølbak K, Skov RL, et al. Increased Transmissibility of SARS-CoV-2 Lineage B.1.1.7 by Age and Viral Load: Evidence from Danish Households. *medRxiv* 2021:2021.04.16.21255459. doi: 10.1101/2021.04.16.21255459

15. Lyngse FP, Mølbak K, Denwood M, et al. Effect of Vaccination on Household Transmission of SARS-CoV-2 Delta VOC. *medRxiv* 2022:2022.01.06.22268841. doi: 10.1101/2022.01.06.22268841

16. Maltezou HC, Vorou R, Papadima K, et al. Transmission dynamics of SARS-CoV-2 within families with children in Greece: A study of 23 clusters. *J Med Virol* 2021;93(3):1414-20. doi: 10.1002/jmv.26394 [published Online First: 20200826]

17. Martinez DA, Klein EY, Parent C, et al. Latino Household Transmission of SARS-CoV-2. *Clin Infect Dis* 2021 doi: 10.1093/cid/ciab753 [published Online First: 20210831]

18. Metlay JP, Haas JS, Soltoff AE, Armstrong KA. Household Transmission of SARS-CoV-2. *JAMA Netw Open* 2021;4(2):e210304. doi: 10.1001/jamanetworkopen.2021.0304 [published Online First: 20210201]

19. Miyahara R, Tsuchiya N, Yasuda I, et al. Familial Clusters of Coronavirus Disease in 10 Prefectures, Japan, February-May 2020. *Emerg Infect Dis* 2021;27(3):915-18. doi: 10.3201/eid2703.203882

20. Park YJ, Choe YJ, Park O, et al. Contact Tracing during Coronavirus Disease Outbreak, South Korea, 2020. *Emerg Infect Dis* 2020;26(10):2465-68. doi: 10.3201/eid2610.201315 [published Online First: 20200716]

21. Rosenberg ES, Dufort EM, Blog DS, et al. COVID-19 Testing, Epidemic Features, Hospital Outcomes, and Household Prevalence, New York State-March 2020. *Clin Infect Dis* 2020;71(8):1953-59. doi: 10.1093/cid/ciaa549

22. Singanayagam A, Hakki S, Dunning J, et al. Community transmission and viral load kinetics of the SARS-CoV-2 delta (B.1.617.2) variant in vaccinated and unvaccinated individuals in the UK: a prospective, longitudinal, cohort study. *Lancet Infect Dis* 2021 doi: 10.1016/s1473-3099(21)00648-4 [published Online First: 20211029]

23. Tibebu S, A. Brown K, Daneman N, et al. Household secondary attack rate of COVID-19 by household size and index case characteristics. *medRxiv* 2021:2021.02.23.21252287. doi: 10.1101/2021.02.23.21252287

24. Wang Z, Ma W, Zheng X, et al. Household transmission of SARS-CoV-2. *J Infect* 2020;81(1):179-82. doi: 10.1016/j.jinf.2020.03.040 [published Online First: 20200410]

25. Wei L, Lv Q, Wen Y, et al. Household transmission of COVID-19, Shenzhen, January-February 2020. *medRxiv* 2020:2020.05.11.20092692. doi: 10.1101/2020.05.11.20092692

26. Wu J, Huang Y, Tu C, et al. Household Transmission of SARS-CoV-2, Zhuhai, China, 2020. *Clin Infect Dis* 2020;71(16):2099-108. doi: 10.1093/cid/ciaa557

27. Yousaf AR, Duca LM, Chu V, et al. A Prospective Cohort Study in Nonhospitalized Household Contacts With Severe Acute Respiratory Syndrome Coronavirus 2 Infection: Symptom Profiles and Symptom Change Over Time. *Clin Infect Dis* 2021;73(7):e1841-e49. doi: 10.1093/cid/ciaa1072

28. Julin CH, Robertson AH, Hungnes O, et al. Household transmission of SARS-CoV-2; a prospective longitudinal study showing higher viral load and transmissibility of the Alpha variant compared to previous strains. *medRxiv* 2021:2021.08.15.21261478. doi: 10.1101/2021.08.15.21261478
