# Supplementary material for: Susceptibility and infectiousness of SARS-CoV-2 in children versus adults, by variant (wild-type, alpha, delta): A systematic review and meta-analysis of household contact studies
Source: PLoS One. 2024 Sep 6;19(9):e0306740. doi: 10.1371/journal.pone.0306740 (PMC11379298; doi:10.1371/journal.pone.0306740)
Supplement: Attachment — Submitted filename: 00_ResponsesToReviewers_Final.docx [file pone.0306740.s004.docx]

Dear Editor,

Thank you for considering our manuscript "Susceptibility and infectiousness of SARS-CoV-2 in children versus adults, by variant (wild-type, alpha, delta): a systematic review and meta-analysis of household contact studies" for publication in PLOS ONE. We appreciate the thoughtful comments from the reviewers and the opportunity to submit a revised version of our work.

As agreed with you we have not rerun the entire search as it would have resulted in too many new publications. We have, however, done the following:

1. We updated all preprint references to peer-reviewed publications where available and removed one study that had significant methodological changes between preprint and publication, which we considered inappropriate (for details see table with point-to-point reply).
2. we removed global specific case and death numbers from the Introduction.
3. we acknowledged the limited number of included delta studies as a limitation and noted that more recent data are perhaps available, although we doubt that they will be of a similar quality as the Danish study that is included in this review. In addition, please note that the objective of this study was to analyze the effect of the variants themselves, i.e. among immune-naïve persons. Since the advent of omicron, its variants and recombinants the same approach is virtually not feasible anymore.
4. Minor editorial changes for consistency and clarity were made (see track changes in revised manuscript).

With these amendments, we believe our work remains a valuable contribution to understanding the relative susceptibility and infectiousness of (immune-naïve) children and adolescents across SARS-CoV-2 variants. Our systematic approach focusing on household studies only and on isolating the effects of each variant independent of vaccination provide a rigorous analysis of this important public health question.

Below, we have provided detailed point-by-point responses to each of the reviewers' comments, outlining the specific changes made or justifications provided for our approach. We hope these will satisfactorily address any outstanding concerns.

Yours sincerely,

Ola Uthman for the Authors

**Point-by-point response to Reviewers’ comments**

| s/n | **Editor’s comments** | Response, not yet finely formulated, just first reaction |
| --- | --- | --- |
|  | Although the Alpha and Delta variants are no longer circulating today, your analysis is of interest as it provides insight into the impact that SARS-CoV-2 has had on its evolution. Throughout the pandemic, the pediatric population has been of particular interest to the medical world due to the response that children have had to the infection. The data you present is of interest and worth publishing. | Thanks for the comments |
|  | I recommend a revision of the text to update it, given that it was written in 2022. | We have checked the referenced preprints to see if they have been published in peer-reviewed journals. Where this is the case, we have updated the references accordingly. Upon review, most of the data presented in the preprints remained the same in the final publications, with one notable exception. In the Cerami et al. study, the overall attack rate changed significantly between preprint and publication (from 60% to 33%) because household contacts who tested positive at the initial baseline were excluded in the final analysis. As we disagree with this post hoc exclusion of early household transmission events, we have decided to remove this study from our review. |
|  | The introduction should be changed without reference to the number of cases and deaths. | We have removed the specific case and death numbers from the Introduction as suggested. It states now:  “Since emergence of SARS-CoV-2, the virus has caused hundreds of millions of COVID-19 cases and millions of deaths^1^.” |
|  | Review the discussion on Omicron and the limitations part where it makes mention of Omicron. | Regarding the comments on omicron and associated limitations, we have reviewed the relevant sections of our discussion and feel they already address these points adequately. Perhaps the delta wave was the last where the "true" effect of a variant alone, irrespective of vaccination and previous infection, was possible in household contact studies as done here. As vaccine uptake became widely available - restricting analysis to unvaccinated persons may introduce a bias due to different adherence and attitudes towards recommended public health and infection prevention measures. In addition, after the omicron wave the majority of the population likely had experienced at least one, not always documented, SARS-CoV-2 infection.  In the text we say: “After omicron and its variants or recombinants began to dominate worldwide such studies have become more difficult to interpret as the vast majority of the population either has been vaccinated various times or had experienced at least one, not always documented, SARS-CoV-2 infection^60^.”  "Although after the advent of VOC omicron analyses restricting to unvaccinated index cases and unvaccinated (and previously uninfected) household contact persons have almost become impossible further household transmission studies are warranted nevertheless but will have somewhat different interpretations compared to this study."  We believe these statements, with their supporting reference, make the key points about the difficulty of assessing intrinsic variant properties in the omicron era, although household transmission studies will continue to be important. We have therefore not made any further edits to this section. |
|  | **Reviewers' comments:** |  |
|  | Reviewer #1: The authors have performed a a systematic reivew of susceptiblity and infectiousness of SARS-CoV-2 in children versus adults by variant type - wild type, alpha and delta. | Thanks. |
|  | The authors defined "household", "index/primary case", "household contact" NB acronym hhc should be capitalised. | We have made the editorial changes suggested by the reviewer and capitalized the 'HHC' abbreviation consistently throughout the manuscript. Regarding the issue of using index or primary case we have elaborated in more detail in Methods: “We referenced in eTable 1 the terms “index case” or “primary case” as used in the studies themselves (**eTable 1**). While these terms have different meanings (index case is usually defined as the first case to be confirmed in a household, primary case is usually the case with the earliest date of symptom onset) for consistency purposes and to avoid confusion we have used the term “index case” for the rest of the paper.” |
|  | Why should vaccinated individuals be excluded as a contact? Very relevant in delta (and more recent variants); and useful to understand the differences in susceptiblity in vaccinated versus unvaccinated individuals. | The reviewer raises the valuable point that understanding susceptibility differences between vaccinated and unvaccinated contacts would be highly relevant and useful, especially for the delta variant and beyond. We agree this is an important question. However, the primary aim of our study was to isolate the effect of each variant in the absence of immunity from vaccination or prior infection. Our inclusion criteria and analyses were specifically designed around this goal. While undoubtedly interesting, directly comparing vaccinated and unvaccinated individuals would require a different study design and research questions.  We have added the following sentence to our discussion of limitations to acknowledge this:  "We did not examine differences in susceptibility and infectiousness between vaccinated and unvaccinated household contacts as our inclusion criteria and analyses were designed to isolate the effects of each SARS-CoV-2 variant independent of immunity from vaccination or prior infection. While this allowed us to assess the intrinsic properties of the variants themselves, it does not capture the real-world impact of vaccines that were widely available during circulation of the Delta variant and beyond. Future household transmission studies should consider stratifying by vaccination status or measure immunity status serologically, as understanding susceptibility and infectiousness differences between vaccinated and unvaccinated individuals is relevant for guiding public health policies and recommendations.”  We hope this addition clarifies the rationale for our study's focus while highlighting the importance of the reviewer's suggested analysis for other research. |
|  | The authors mention that a large proportion of studies were from China and the USA, but would recommend mentioning whether all studies were performed in high-moderate income countries. As this is a systematic review, the authors are behest to the quality of the studies. That said, it is important for the reader to understand and would recommend the authors to comment on 1. average household size, 2. guidance on isolation away from household members (or note it as a limitation if unknown) as this will affect both susceptibility and infectiousness. | We appreciate the reviewer's suggestion to comment on the geographic and economic settings of the included studies and how these factors may impact household transmission dynamics.  Regarding point 1, we have added the following statement to the Discussion section:  "As the majority of studies were conducted in high- or middle-income countries, with 8 studies (30%) from China and 6 studies (22%) from the United States. Household sizes in these settings may differ from those in low-income countries, potentially affecting secondary attack rates."  Regarding point 2, we agree that variation in guidance on isolation from household members is an important consideration when interpreting household transmission data. However, systematically examining isolation policies and practices across all included studies and settings was outside the scope of our review. This information was often not reported in the primary studies, and even when guidance was mentioned, actual implementation within households may have varied considerably. Nevertheless, please note that we have – unlike other reviews – focussed on household studies alone in order to improve comparability of the data.  At any rate to acknowledge the limitation that guidance how to deal with household contacts exposed to an index case was not taken into account, we have added the following paragraph to the Discussion:  "Another potential limitation is that we did not assess differences across studies in guidance on isolation of infected individuals to protect household members. Such guidance likely varied between and within countries over time, and adherence within households may have been inconsistent and would have to be recorded also. Stricter isolation policies could reduce household transmission, while limitations in household space or resources could make effective isolation difficult. As long as household studies are not standardized this type of information will not be available in an analysable way. Only randomized trials assigning households to different isolation protocols could provide more definitive evidence, but such designs may be logistically and ethically challenging." |
|  | The authors mention that the search was conducted up to and including January 2022, and therefore only 1 delta household study has been included. I think the authors should acknowledge that further studies have been published, in other settings to better inform HH transmission of the delta variant. | The reviewer correctly notes that our literature search only extends to January 2022, resulting in only a single included study for the Delta variant. They suggest we should acknowledge that additional relevant delta studies have likely been published since then.  We have added the following statement to the Discussion section on limitations:  "Our systematic review includes literature published up to January 2022, and as such only one study on the delta variant met our inclusion criteria. It is possible that additional household transmission studies of the delta variant have been published. The single included (Danish) study may not fully capture the heterogeneity of delta transmission across different settings and populations. Having said that we believe that not many study groups will have been able to control for vaccination and previous infections and with the same statistical power as has been done in the cited Danish study which is included in this review."  This addition recognizes the limited delta data as a constraint of our search strategy while still emphasizing the strength of the included study. We believe this is the most transparent and balanced approach given the limitations inherent in updating our analysis, as detailed in the cover letter. |
|  | Variants are not usually capitalised (e.g. should read alpha, delta, omicron etc) | We have revised all generic mentions of variants to use lowercase letters. |
|  | Page 16 - consider revising "After the omicron such studies have..." (remove 'the') | After omicron and its variants or recombinants began to dominate worldwide such studies have become more difficult to interpret… |
